# Senescence as a novel mechanism involved in β-adrenergic receptor mediated cardiac hypertrophy

**DOI:** 10.1371/journal.pone.0182668

**Published:** 2017-08-04

**Authors:** Rongrong Sun, Baoling Zhu, Kai Xiong, Yan Sun, Dandan Shi, Li Chen, Youyi Zhang, Zijian Li, Lixiang Xue

**Affiliations:** 1 Department of Biochemistry and Molecular Biology, Peking University Health Science Center, Beijing, China; 2 Institute of Vascular Medicine, Peking University Third Hospital, Beijing, China; 3 Medical Research Center, Peking University Third Hospital, Beijing, China; 4 Department of Radiation Oncology, Peking University Third Hospital, Beijing, China; Texas A&M University Health Sciences Center, UNITED STATES

## Abstract

Pathological cardiac hypertrophy used to be elucidated by biomechanical, stretch-sensitive or neurohumoral mechanisms. However, a series of hints have indicated that hypertrophy process simulates senescence program. However, further evidence need to be pursued. To verify this hypothesis and examine whether cardiac senescence is a novel mechanism of hypertrophy induced by isoproterenol, 2-month-old male Sprague Dawley rats were subjected to isoproterenol infusion (0.25mg/kg/day) for 7 days by subcutaneous injection). Key characteristics of senescence (senescence-associated β-galactosidase activity, lipofuscin, expression of cyclin-dependent kinase inhibitors) were examined in cardiac hypertrophy model. Senescence-like phenotype, such as increased senescence-associated β-galactosidase activity, accumulation of lipofuscin and high levels of cyclin-dependent kinase inhibitors (e.g. p16, p19, p21 and p53) was found along the process of cardiac hypertrophy. Cardiac-specific transcription factor GATA4 increased in isoproterenol-treated cardiomyocytes as well. We further found that myocardial hypertrophy could be inhibited by resveratrol, an anti-aging compound, in a dose-dependent manner. Our results showed for the first time that cardiac senescence is involved in the process of pathological cardiac hypertrophy induced by isoproterenol.

## Introduction

Pathological cardiac hypertrophy is the cellular response to biomechanical or neurohumoral stimuli. The defining features of hypertrophy are increased cardiomyocyte size, enhanced protein synthesis and reinduction of the so-called fetal gene program. Although hypertrophy has traditionally been considered as an adaptive response required to sustain cardiac output, in the long term, hypertrophy predisposes individuals to heart failure, arrhythmia and sudden death [1, 2]. Despite the recent advances in understanding the molecular and cellular processes that contribute to cardiac hypertrophy [2–4], there remains large unknown and the need for further investigation.

Cellular senescence was first introduced by Hayflick and Moorhead [5] to describe the permanent form of cellular proliferative arrest. Senescent cells are characterized by phenotypic changes[5–9]; for example, increased cell size, enhanced senescence-associated β-galactosidase (SA-β-gal) activity at pH 6 and high levels of cyclin-dependent kinase inhibitors (CDKIs), e.g.p16^INK4a^, p21, p53 et, which block the cell cycle. The mammalian heart has long been considered a quiescent organ. Although there are a few studies suggesting that cardiomyocytes can divide at a low rate under certain conditions [10, 11], it is widely believed that the majority of cardiomyocytes, if not all of them, are out of cell cycle shortly after born. Therefore, the question that has been raised is whether cardiomyocytes can undergo senescence. Previous studies[12, 13] have revealed that cardiomyocytes from old mice show certain senescence-associated properties, including high SA-β-gal activity, increased CDKIs expression, accumulated lipofuscin and decreased telomerase activity. Senescence-like features have also been reported for post-mitotic neurons from old C57Bl/6 mice [14] and adipocytes of mice on a high-fat diet [15], suggesting that post-mitotic cell senescence might be a broader phenomenon.

Aging is an independent risk factor of cardiovascular diseases. Hearts of aged mice and human showed hypertrophy and fibrosis [12, 16, 17]. Besides, cellular senescence and cardiac hypertrophy share certain features [1, 18]: an increase in cardiomyocyte size and enhanced protein synthesis. In addition, activation of β-adrenergic receptor (β-AR) signaling is one of the most important pathophysiological mechanisms of cardiac hypertrophy. Interestingly, recent researches established a role for β-AR signaling in mammalian longevity. Yan et al. reported that mice lacking *ADCY5*, encoding type 5-adenylyl cyclase (AC5) which activates the signaling transduction of β-AR, are stress resistant and have experienced a 30% increase in median lifespan [19]. On the other hand, transgenic mice engineered to overexpress β2-AR in cardiac tissue have reduced lifespan. In support, enhanced production of β2-AR caused by genetic variants is inversely associated with human lifespan [20]. Based on the fact that cardiac senescence and hypertrophy share defining features and signaling pathways, the aim of our study is to find out whether cardiac senescence is involved in the process of pathological cardiac hypertrophy and what could be the specific biomarkers for evaluating cardiac aging.

## Materials and methods

### Animals

2-month-old (300-350g) and 24-month-old (700-850g) male Sprague Dawley rats were purchased from the Animal Center of Tianqin, Changsha. Rats were raised in SPF environment at room temperature (25±2)°C and provided with a standard diet and water in compliance with the Institutional Animal Care and Use Committee of Peking University Health Science Center.

### Isoproterenol-induced cardiac hypertrophy model

Cardiac hypertrophy induced by isoproterenol (ISO) was generated as previously described [21, 22]. ISO (0.25mg/kg/day) was administered to 2-month-old rats for 7 days by subcutaneous injection). Identical volume of saline was administered to age-matched rats as control. All protocols were approved by the Institutional Animal Care and Use Committee of Peking University Health Science Center.

### Echocardiography

24h after the last administration, trans-thoracic echocardiography was performed on rats with 2.0% isoflurane using a Vevo 770 ultrasound machine (VisualSonics, Toronto, Canada) with a 30 MHz probe (RMV707B). Two-dimensional echocardiography was captured by a short axis view at the level of the papillary muscles for the largest LV diameter. The diastolic left ventricular posterior wall thickness (LVPW;d) was measured from M-mode tracings. The average of three consecutive cardiac cycles was taken for each parameter. Echocardiography procedures were operated in accordance with the guideline of American Society of Echocardiography.

### Histology analysis

For histological analysis, part of heart tissues were fixed in 4% paraformaldehyde (PFA) at 4°C overnight, then dehydrated and embedded in paraffin for preparation of 5-μm histological sections. Rehydrated slides were stained with haematoxylin and eosin (HE) staining to evaluate cardiomyocyte area and picric-sirius red staining to measure fibrosis area. Another part of heart tissues were fixed in 20% sucrose solution at 4°C overnight and embedded in O.C.T. compound for preparation of 8-μm frozen sections. Detection of senescent cells was determined in cells and frozen sections with the senescence β-galactosidase staining kit (GenMed Scientifics Inc.U.S.A) and lipofuscin staining kit (GenMed Scientifics Inc.U.S.A). Samples were analyzed by two independent investigators in a blind fashion.

### Protein analysis

Total protein was extracted from frozen heart, resolved and electrotransferred as described. Antibodies used for western blot were as follows: anti-p16 (Abcam), anti-p19 (MBL International Corporation), anti-p21 (MBL International Corporation), anti-p53 (MBL International Corporation), anti-GATA4 (Santa Cruz Biotechnology), and anti-eif5 (Santa Cruz Biotechnology).

### Quantitative RT-qPCR

Total RNA was extracted from heart tissue using Trizol^®^ Reagent (Invitrogen), and cDNA was synthesized using M-MLV reverse transcriptase (Takara Bio) and random primers (Takara Bio) according to manufacturer instructions. Quantitative PCR was performed in triplicate using the Gene Expression Assay (Applied Biosystems) on an Applied Biosystems Fast 7500 machine with GAPDH as an endogenous normalization control. Primer sequences are available upon request.

### Cell culture and treatment

Neonatal cardiomyocytes (NRCM) from 1- or 2-day-old SD rats were isolated as described previously. Cardiomyocytes were incubated in DMEM supplemented with 10% FBS for 36 h at 37°C. After subjected to serum free culture for 12h, NRCM were pre-incubated with 10^−6^, 10^−5^ and 10^−4^ mol/L resveratrol for 30min. Then 10^-6^mol/L ISO was added to the culture medium containing resveratrol for 48h [23, 24]. To visualize cardiomyocyte borders, fixed cells were incubated in wheat germ agglutinin conjugated to Alexa Fluor 488 (Invitrogen) at 1 mg/ml in PBS. Nuclei were stained blue by DAPI. All experiments were performed in accordance with the Guide for the Care and Use of Laboratory Animals and approved by the Institutional Animal Care and Use Committee of Peking University Health Science Center University.

### Statistical analysis

GraphPad Prism Software was used for data analysis. All data are expressed as the mean±SEM. Paired data were evaluated using Student’s *t*-test. Differences were considered statistically significant at P<0.05.

## Results

### The establishment of pathological cardiac hypertrophy model

**Fig 1:**
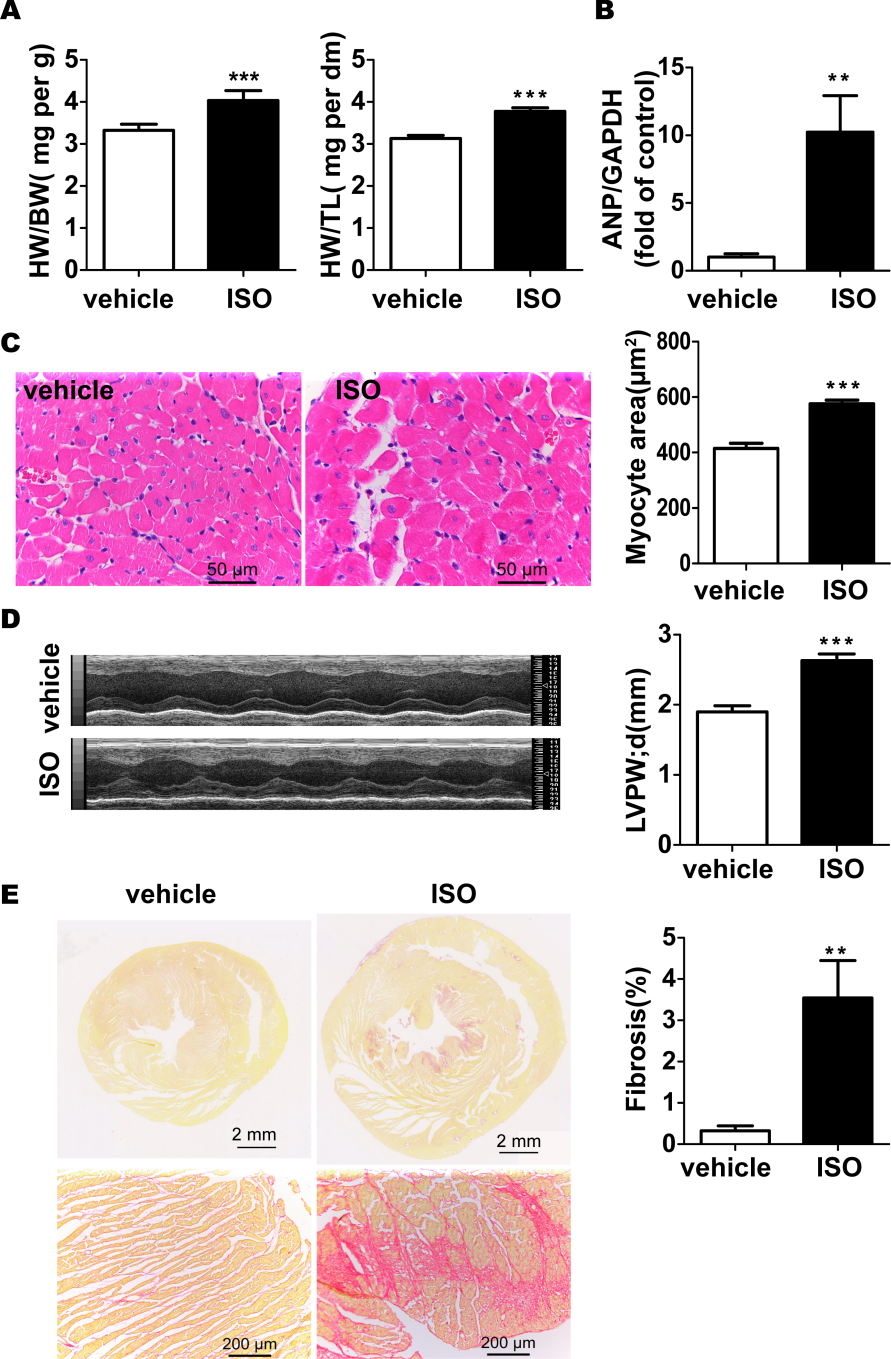
Pathological cardiac hypertrophy induction. (A) HW/BW and HW/TL ratio in ISO-treated rats and controls (B) The gene expression of ANP was examined with the use of quantitative RT-PCR. (C) Cardiomyocyte area was evaluated by H&E staining and quantified. (D) Wall thickness was measured from M-mode tracings as described in the methods and calculated. (E) The percent area of myocardial fibrosis was examined by picric-sirius red staining. Data are means ± SEM (n = 6; **P < 0.01, ***P < 0.001 *vs*. vehicle group).

To analyze whether cardiac senescence is involved in the process of pathological cardiac hypertrophy, we first induced hypertrophy in 2-month-old rat hearts by infusion with ISO. Canonical hypertrophic markers, such as heart-to-body-weight (HW/BW) and heart-weight-to-tibia-length (HW/TL) ratio, cardiomyocytes size and expression level of atrial natriuretic peptide (ANP), was selected to assess cardiac hypertrophy model. After subcutaneous injection of ISO for 7 days, SD rats had higher HW/BW (Fig 1A) and HW/TL (Fig 1A) ratio compared with controls. This data was further confirmed by HE staining, which showed enlarged cardiomyocytes area. Moreover, these hearts had markedly thicker ventricular wall and expressed higher level of ANP than controls (Fig 1B–1D). Fibrosis, as the main distinction between pathological and physiological cardiac hypertrophy, is also found marked increase in rats upon ISO treatment (Fig 1E). Taken together, these results indicate that the model of pathological cardiac hypertrophy was successfully established by ISO in rats.

### Increased number of SA-β-gal positive cardiomyocytes and amount of lipofuscin in ISO-treated cells

**Fig 2:**
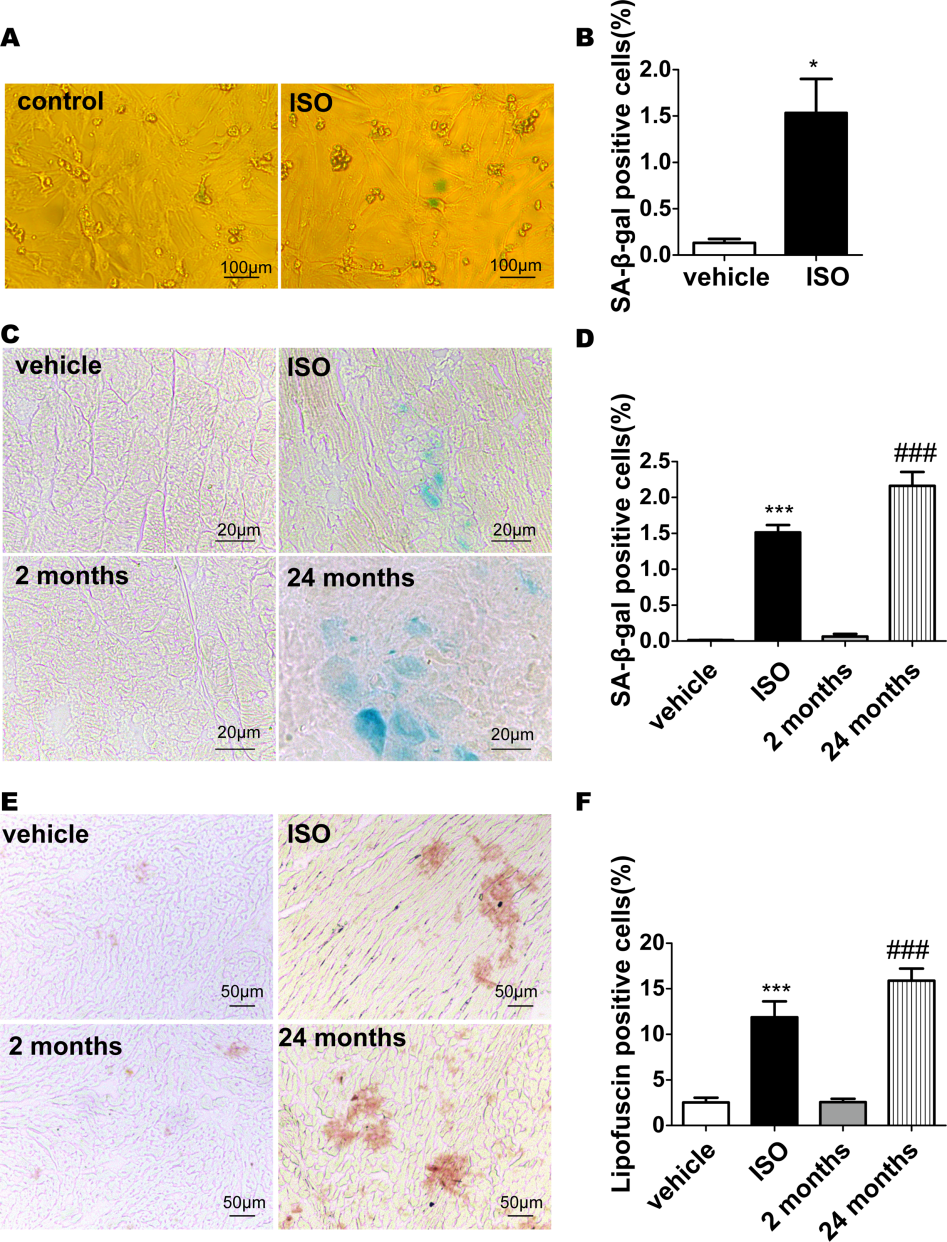
Increased number of senescence associated β-galactosidase (SA-β-gal)-positive cardiomyocytes and amount of lipofuscin in aging and treated cells. (A) Cardiomyocytes were incubated with 10^−5^ mol/L ISO for 48h. Cells were then stained for the presence of SA-β-gal as described in the Methods. Data are means ± SEM (n = 3; *P < 0.05 *vs*. vehicle). (B) The percentage of SA-β-gal-positive cells was calculated. (C) Frozen heart tissue sections were analyzed for SA-β-gal staining, and (D) the number of SA-gal-positive cells was counted. (E), Frozen heart tissue sections were analyzed for lipofuscin and (F) the percentage of lipofuscin-positive cells was calculated. Data are means ± SEM (n = 6; ***P < 0.001 vs.vehicle group; ###P < 0.001 *vs*. 2 months.).

To verify our hypothesis that whether senescence is involved in the hypertrophy process, key characteristics of senescent cells were examined in the cardiac hypertrophy model described above. We first examined SA-β-gal activity in cultured neonatal rat cardiomyocytes incubated with 10^−6^ mol/L ISO for 48h. The percentage of SA-β-gal positive cells increased significantly compared with that of the control cells (control: 0.133%±0.043%; ISO: 1.533%±0.367%; n = 3, p<0.05, Fig 2A and 2B). We further detected SA-β-gal activity in tissue sections from ISO induced hypertrophy rat model and found the positive staining cardiomyocytes were rarely observed in the myocardium of vehicle group, whereas the ratio of SA-β-gal positive cardiomyocytes was significantly increased upon ISO treatment (vehicle: 0.013%±0.005%; ISO: 1.514%±0.101%, n = 6, p<0.001; Fig 2C and 2D). As the parallel positive control, SA-β-gal staining was also assessed in old rats without any treatment (Fig 2C). SA-β-gal positive cells in young (2-month-old) rats comprised 0.064%±0.036%, in aged (24-month-old) rats comprised 2.163%± 0.193% (n = 6; P < 0.001; Fig 2D) which is comparable to the cardiomyocytes in ISO-induced hypertrophy model. These results indicate that the standard aging biomarker, SA-β-gal activity, is enhanced in ISO-treated cardiomyocytes.

Another highlight for cellular senescence is the intracellular accumulation of lipofuscin, especially for post-mitotic cells which are not capable to degrade or get rid of it. Lipofuscin is known as an autofluorescent, nondegradable, and polymeric substance composed primarily of cross-linked protein and lipid residues [25, 26]. Previous studies showed that cardiomyocytes of aged hearts accumulate a large amount of lipofuscin due to imbalance between protein damage and clearance of damaged proteins[25]. To investigate whether cardiac hypertrophy model induced by ISO exhibited a senescent phenotype, the amount of lipofuscin was examined. Compared with age-matched (2-month-old) vehicle group, frozen sections of rats treated with ISO accumulated a large amount of lipofuscin (vehicle: 2.533%±0.533%; ISO:11.880%±1.747%; n = 6, p<0.001, Fig 2E and 2F). This phenomenon was also observed in lipofuscin staining of aged (24-month-old) rats as the positive control (2-month-old rats: 2.583%±0.354%; 24-month-old:15.900%±1.309%, n = 6; p<0.001; Fig 2E and 2F). These results indicate that lipofuscin, another senescent marker, accumulated in ISO-treated cardiomyocytes.

### Expression of CDKIs and GATA4 increased in ISO-treated cells

**Fig 3:**
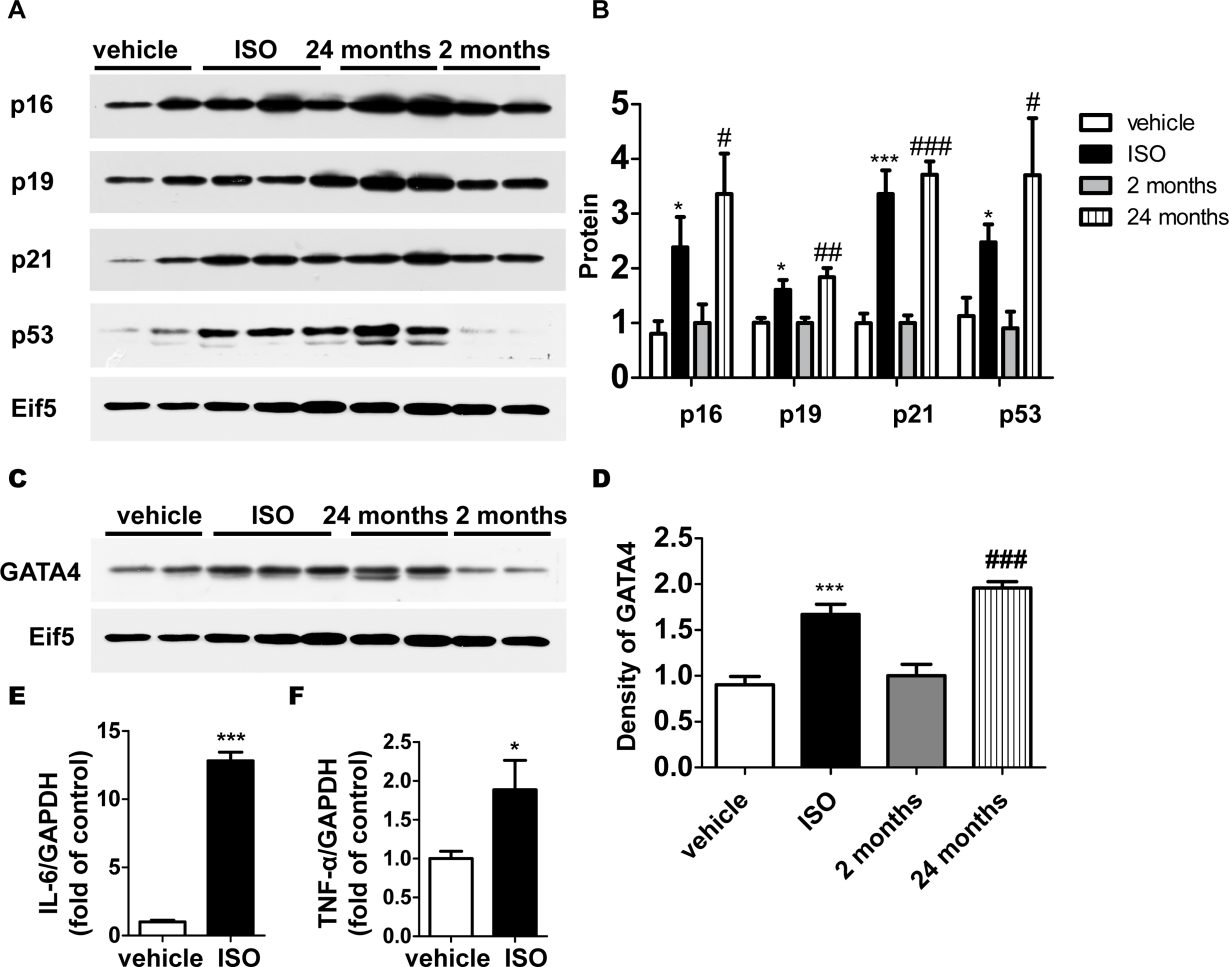
Expression of CDKIs and GATA4 increased in ISO-treated cells. (A) The protein level of cell cycle inhibitors and Eif5 (loading control) was examined by western blotting. (B) The protein level was quantified by densitometry. (C) The protein level of GATA4 and Eif5 was examined by western blotting and (D) quantified by densitometry. The gene expression of SASP factors (E) and (F) was examined with the use of quantitative RT-PCR. Data are means ± SEM (n = 6; *P < 0.05, ***P < 0.001 *vs*. vehicle; # P < 0.05, ## P < 0.01,###P < 0.001 *vs*. 2 months. The mean value for 2-month-old rats was expressed as 1 unit).

Increased level of CDKIs, which block the cell cycle is one of the most classic mechanisms of mitotic cell senescence [7, 8, 27, 28]. Since Diana Jurk et al[14] for the first time testified that post-mitotic neurons develop a p21-dependent senescence-like phenotype driven by a DNA damage response in vivo, the role of CDKIs in post-mitotic cell senescence has got more and more attention. The gene expression of CDKIs was evaluated to confirm the involvement of cardiac senescence in hypertrophy process. The results of western blot demonstrated that gene expression of p16, p19 and p21 significantly increased in ISO-treated rats compared with that of age-matched (2-month-old) vehicle group, as well as p53 (Fig 3A and 3B). Higher protein level of CDKIs in aged (24-month-old) rats as positive control was consistent with previous reports [12, 13, 29]. The similar expression pattern of high expressed CDKIs in cardiomyocyte not only showed the senescent phenotype, but also indicated that CDKIs might have some other potential function beyond the cell-cycle regulation in hypertrophy.

Besides applying canonical aging biomarkers, we are trying to find some cardiac-specific hints. GATA4 plays a key role in cardiac specification, development and function. Perturbation of transcription factor expression and regulation disrupts normal heart structure and function [30–32]. The recent research described that GATA4 is stabilized in cells undergoing senescence and in turn activates the transcription factor NF-κB to initiate the SASP and facilitate senescence. Therefore, GATA4 might be considered as a positive senescence regulator [33]. To examine whether GATA4 functions in cardiac senescence, western blot was performed to detect the expression of GATA4 in cardiomyocytes. The results showed the expression of GATA4 was up-regulated in hearts of aged (24-month-old) rats compared with 2-month-old rats (Fig 3C and 3D). This phenomenon was also observed in ISO-treated cardiomyocytes (Fig 3C and 3D). SASP genes were also examined by realtime PCR. The mRNA level of interleukin-6(IL-6) and tumor necrosis factor-α (TNF-α) both increased in ISO-treated group (Fig 3E and 3F). These results not only present the similar phenotype between hypertrophy and cardiac senescence, but also indicate that the transcription factor GATA4 might be actively involved in heart aging and hypertrophy. The novel function of GATA4 in aging need to be further explored.

### Resveratrol prevents pathological cardiac hypertrophy induced by ISO

**Fig 4:**
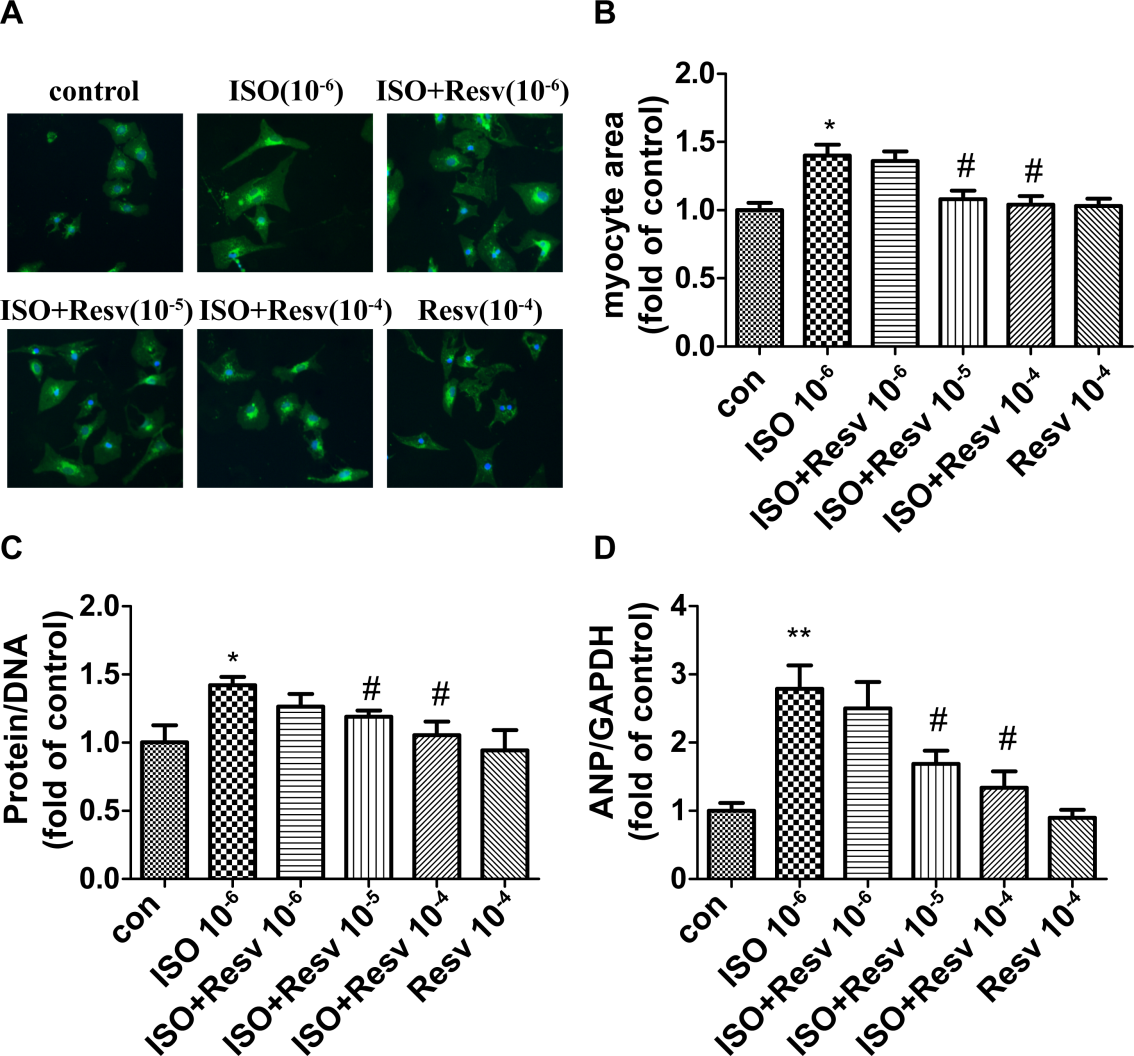
Resveratrol prevents pathological cardiac hypertrophy induced by ISO. Cardiomyocyte area was evaluated by (A) wheat germ agglutinin (WGA) staining and (B) quantified. (C) The ratio of protein/DNA was quantitated. (D) The gene expression of ANP was examined with the use of quantitative RT-PCR. Data are means ± SEM. (n = 3,*P < 0.05, **P < 0.01 *vs*. control; #P < 0.05 *vs*. ISO group. The mean value for control was expressed as 1 unit).

Resveratrol, a phytoalexin, obtained from grape skin possesses diverse biochemical and physiological properties, including antioxidant, antiplatelet, and anti-inflammatory properties as well as a wide range of health benefits ranging from chemoprevention to extending life span[34, 35]. One of the highlights is that resveratrol can mimic the effect of caloric restriction and anti-aging. In order to further confirm the involvement of cardiac senescence in the process of hypertrophy, resveratrol, a widely recognized anti-aging regent was used to verify whether hypertrophy could be intervened upon ISO treatment. It showed that cardiomyocytes treated by ISO possesses the enhanced SA-β-gal activity, while the positive staining percentage decreased in the cardiomyocytes treated by resveratrol in a dose-dependent manner (S1 Fig). In addition, cardiomyocytes stimulated by ISO exhibited typical features of myocyte hypertrophy: enlarged cell area, a higher protein /DNA ratio and highly expressed ANP. While all these phenotypes mentioned above (Cell area, protein/DNA ratio and expression level of ANP) were attenuated by resveratrol in a dose-dependent manner (Fig 4), which suggested cardiomyocyte hypertrophy could be inhibited by resveratrol, thus indicating the senescence mechanism might be involved in this pathological process.

## Discussion

Cellular senescence has been considered as the intrinsic mechanism for tumor suppression. Recently, the emerging evidence shows that senescence progress might be involved in multiple physiological and pathological process besides tumor, such as atherosclerosis and inflammation. Therefore, senescence could be a wider mechanism rather than its phenotype. Moreover, whether senescence also exists in post-mitotic cells such as cardiomyocytes still remains debate. Our present results show for the first time that cardiac senescence phenotype occurs in ISO-induced pathological cardiac hypertrophy by analysis of a wide range of senescence markers. The similar results were also reported in angiotensin II-induced cardiac hypertrophy model and dilated cardiomyopathy caused by cardiac-specific Bmi1 deletion [36] manifested by the increased ratio of SA-β-gal positive cells. It suggested that not only the cardiac senescence does exist in heart but also is involved in multiple hypertrophy models. Increased accumulation of lipofuscin in heart is one of the most consistent features of aging across species of mammals, which is also seen in ISO-treated rats. The mechanism by which lipofuscin accumulates in ISO-induced cardiac hypertrophy model is currently unclear. It was reported that β-AR stimulation provokes cardiac oxidative stress. Especially, in the chronic phase of ISO infusion, ROS may participate in cardiac remodeling, especially in respect to wall stiffness, based on fibrogenesis [37, 38]. Besides, myocyte autophagy was decreased during cardiac hypertrophy, which was associated with progressively increased cardiac oxidative stress [39, 40]. Thus, increased oxidative stress and low autophagy activity lead to the disturbances of proteostasis and an impairment of the proteasomal system which is resulted in the accumulation of highly cross-linked undegradable aggregates such as lipofuscin as the final consequence. Lipofuscin may account in part for the cardiac dysfunction in aged rats and ISO-treated rats due to deleterious effects of lipofuscin on cardiac cellular function. There are several researches showed that post-mitotic cell senescence is associated with activation of inhibitory cell cycle regulators [12, 13]. It was reported that p21 gene expression increased significantly in angiotensin II-induced cardiac hypertrophy [36]. In our study, along the process of hypertrophy, the expression of p21 and p16 increased, as noted in other studies [13]. Notably, p21 is a necessary signal transducer between DNA damage response and senescence-like phenotype in neurons, as in senescing fibroblasts[14]. Increased expression of p21 in ISO-treated cardiomyocytes may due to DNA damage response caused by oxidative stress in the process of cardiac hypertrophy.

Myocyte hypertrophy was suppressed by resveratrol in a dose-dependent manner, which suggested hypertrophy could be inhibited by intervening aging process. In support of our hypothesis, other anti-aging regents, such as rapamycin[41, 42] and metformin[43], are proven to prevents cardiac hypertrophy induced by various pathological stimulants[44–47]. Rapamycin attenuated cardiac hypertrophy mainly by three pathways (a) AKT/mTOR/S6 kinase signaling, which is important in the regulation of protein synthesis[44, 48], (b) promoting autophagy through a mechanism involving the modulation of Noxa and Beclin-1 expression by the MEK/ERK signaling pathway[49] or (c) inhibiting NF-κB activation[50]. Recent studies demonstrated that the anti-hypertrophic effects of metformin are associated with AMPK activation and prevention of mitochondrial dysfunction through the SIRT1/eNOS/p53 pathway [47, 51, 52]. Collecting all these evidence mentioned above, it suggests that targeting anti-aging pathway might become an effective strategy in intervening hypertrophy.

In addition, our study challenges the conventional opinion that senescence is only defined in proliferating cells. From this point of view, we might need to revisit the concept of senescence. The widely accepted category of senescence is divided into replicative senescence and premature senescence. In this study, 2–3 month old rats were used to induce myocardial hypertrophy, which exhibited senescence-like features. Such changes did not completely simulate physiological aging process. The most notable difference in our study is that cardiac systolic function did not show the difference between 2-month and 24-month old rats in physiological aging as reported[12], while cardiac systolic function enhanced after 7 days ISO treatment in pathological aging (S2 Fig). This phenomenon may be partially related to the compensation in the long process of physiological aging. While, enhanced cardiac systolic function might due to positive inotropic action of ISO at the early time point (day 7) of administration. According to our study, cardiac systolic function declined at the late time point upon administration (day 14) in C57BL/6 mice (unpublished data). Meanwhile, there is no clear definition for heart aging so far. Since the widely accepted category of senescence is divided into replicative senescence and premature senescence, likewise, cardiac senescence might be subdivided into myocardial physiological aging and pathological aging. Nevertheless, the further investigation on mechanism of cardiac senescence is necessary.

Effective biomarkers are crucial to assess aspects of aging. SA-β-gal staining, expression of CDKIs and SASP factors, lipofuscin and telomere foci are included in the markers which have been applied in cardiac senescence [12, 13, 53]. Although each of these markers is related to senescence, the effectiveness varies. For example, the percentage of SA-β-gal staining in aged heart is extremely low compared with other tissues in the same rat (S3 Fig**)**, such as liver, due to the tremendous difference in the base level of SA-β-galactosidase in each specific tissues [8]. In addition, most of SASP factors are acute phase reactants that exhibit a marked change in expression in response to viral infection and other intercurrent illness unrelated to aging[8]. Therefore, it is valuable to explore organ-specific even cell-specific senescence biomarkers other than conventional markers for the cardiac senescence. Previous study [33, 54] reported that the key transcriptional factor during heart development, GATA4, plays an important role in senescence by activating the transcription factor NF-κB to initiate the SASP and facilitate senescence phenotype. Moreover, there is a significant spatial correlation between GATA4 and p16^INK4a^ in oligodendrocytes, pyramidal neurons, and astrocytes from older humans, further supporting the role of GATA4 in senescence during human aging. In our study, GATA4 is upregulated in ISO-induced hypertrophy model and 24-month old rats, which could serve as an indicator for the heart aging. Although the specificity of GATA4 in heart aging is still need to be further investigated, it somewhat directs the future research in exploring the markers to identify the aging and hypertrophy.

**Fig 5:**
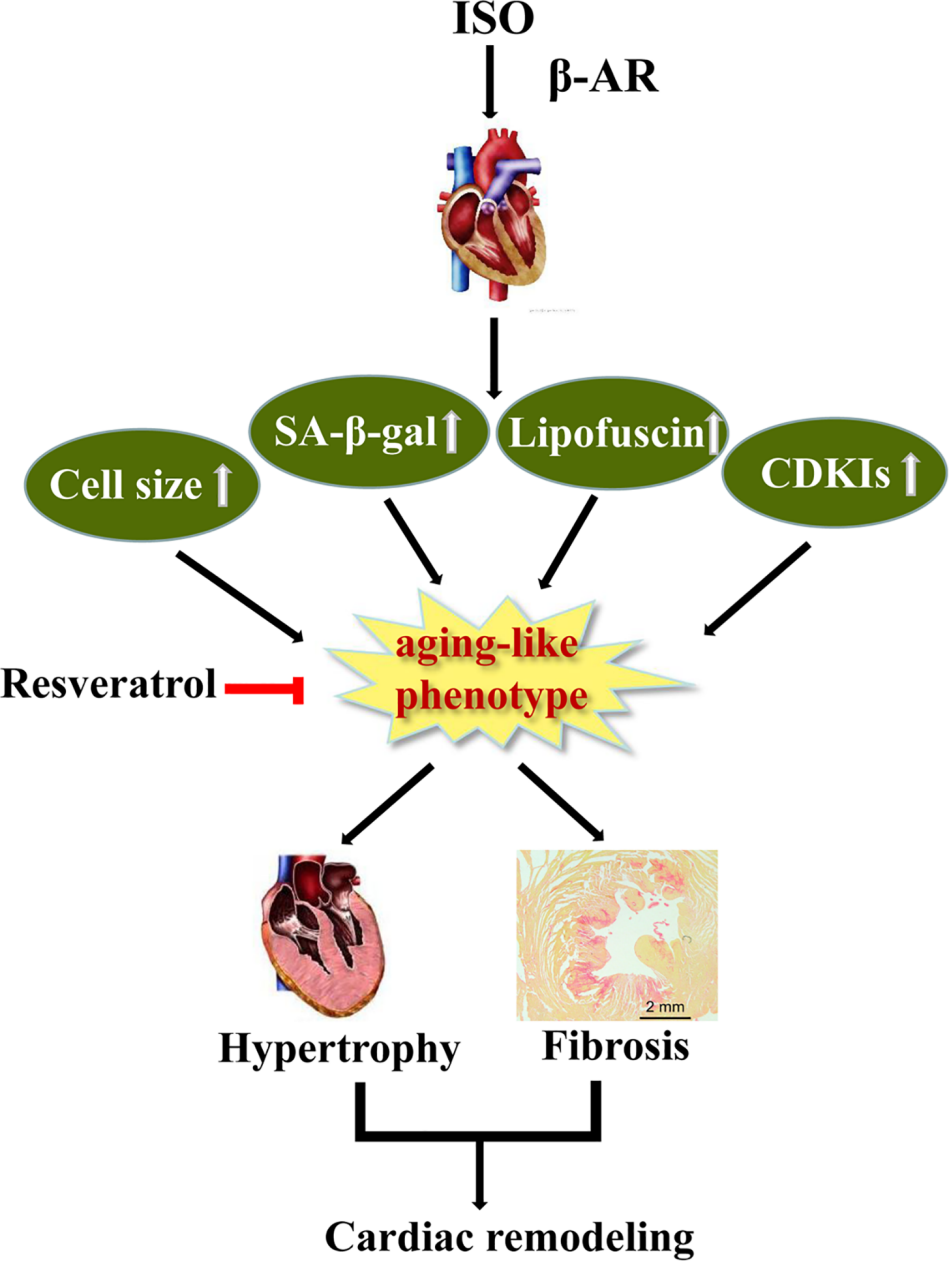
A working model for the senescence mediated β-AR induced cardiac hypertrophy.

Aging is an important risk factor of cardiovascular diseases such as hypertension, cardiac hypertrophy and heart failure. In our study, we also detected several parameters showing hearts of aging rats present cardiac hypertrophy (S4 Fig**)**. Accordingly, senescence mechanism might contribute to promoting a certain diseases progress. Thus, our study might provide a possible prospect that anti-aging might be a powerful strategy for treating cardiovascular diseases, at least for pathological hypertrophy (Fig 5). Indeed, along the same lines, several studies showed anti-aging reagent does help in certain types of diseases, such as rapamycin was proven to prevents cardiac hypertrophy induced by various pathological stimulants [44, 48–50]. The more precise relationship between hypertrophy and senescence still need to be further dissected, and the intensive mechanism is still required to be further explored as well, such as the impact of microenvironment between fibroblast and cardiomyocyte, the sequential and/or interplay effect between these two types of cell in heart during the aging process. These findings will ultimately shed light on the cardiac aging and related diseases.

## Supporting information

S1 Fig SA-β-gal activity was decreased in the cardiomyocytes treated by resveratrol in a dose-dependent manner. (A) Cardiomyocytes were stained for the presence of SA-β-gal as described in the Methods. (B) The percentage of SA-β-gal-positive cells was calculated. Data are means ± SEM (n = 3; *P < 0.05 vs.control group,# P < 0.05 *vs*.ISO group).
(TIF)

S2 Fig (A) Ejection fraction and (B) fractional shortening in 2-month-old and 24-month-old rats; (C) ejection fraction and (D) fractional shortening in ISO-treated rats and controls. Data are means ± SEM, n = 6, **P < 0.01 *vs*. vehicle group.
(TIF)

S3 Fig SA-β-gal staining of different tissue samples between young and old. Frozen sections of heart, liver, spleen, lung and kidney from young (2-month-old) and old (24-month-old) rats were analyzed for SA-β-gal staining (n = 6).
(TIF)

S4 Fig Hearts of aged rats showed hypertrophy. (A) HW/TL ratio in 2-month-old and 24-month-old rats. Cardiomyocyte area was evaluated by H&E staining(C) and quantified (B). The gene expression of β-MHC (D) and ANP (E) were examined with the use of quantitative RT-PCR. Data are means ± SEM, n = 6, # P < 0.05, ###P < 0.001 *vs*. 2 months.
(TIF)
