# Supplementary figures and images for: Senescence as a novel mechanism involved in β-adrenergic receptor mediated cardiac hypertrophy
Source: PLoS One. 2017 Aug 4;12(8):e0182668. doi: 10.1371/journal.pone.0182668 (PMC5544424; doi:10.1371/journal.pone.0182668)

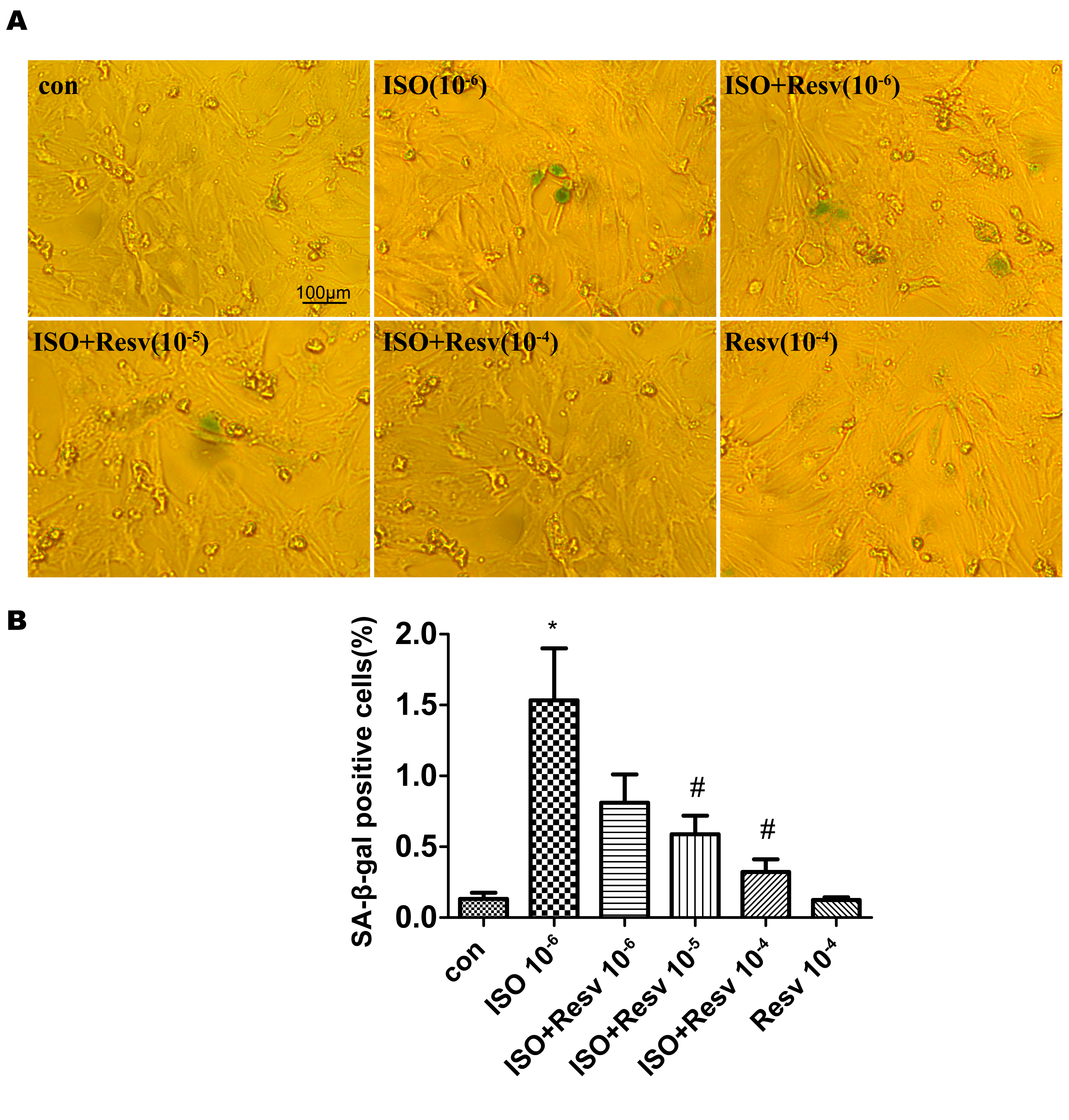

Supplement: S1 Fig — (A) Cardiomyocytes were stained for the presence of SA-β-gal as described in the Methods. (B) The percentage of SA-β-gal-positive cells was calculated. Data are means ± SEM (n = 3; *P < 0.05 vs.control group,# P < 0.05 vs.ISO group). (TIF) [file pone.0182668.s001.tif]

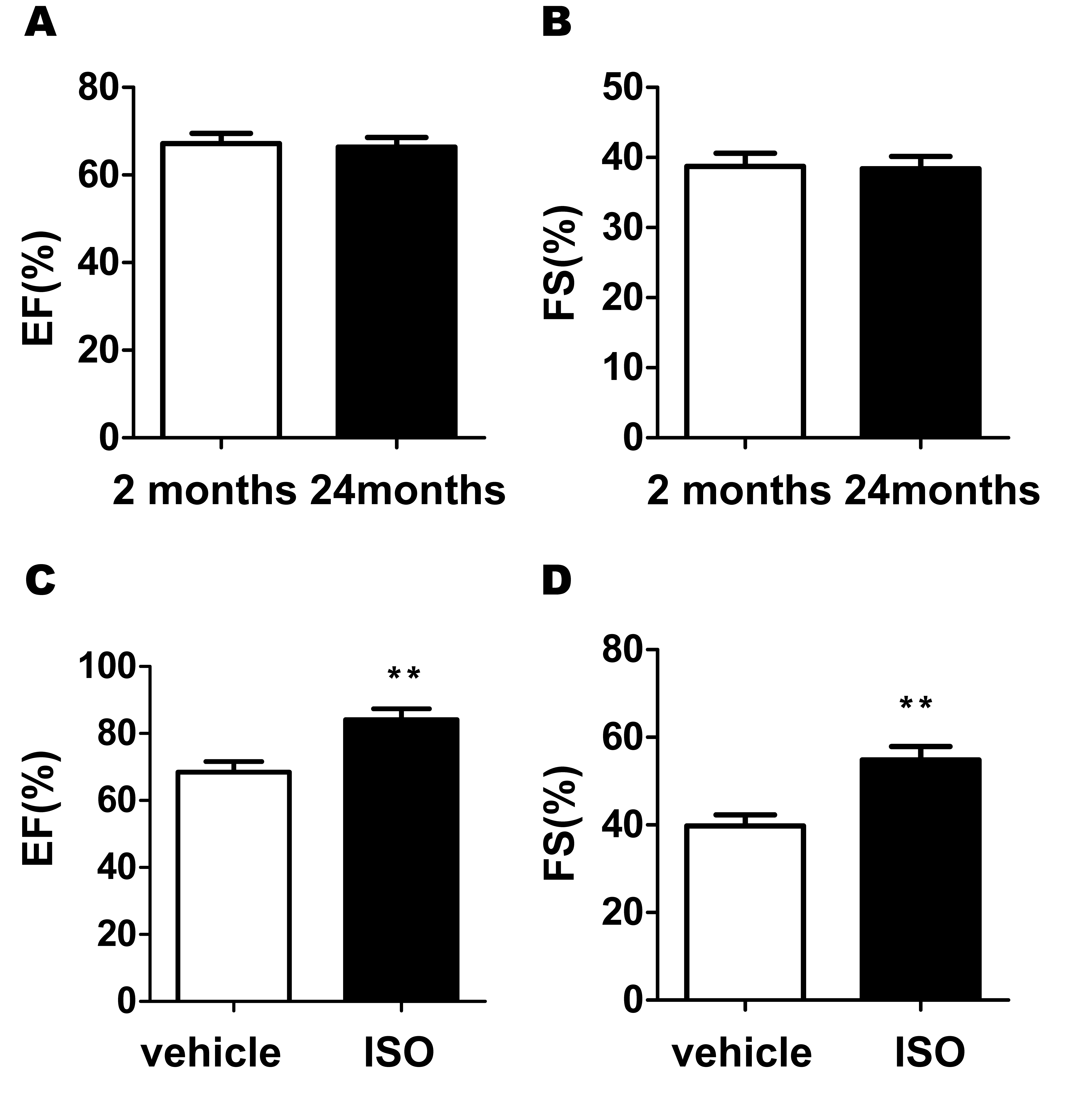

Supplement: S2 Fig — (A) Ejection fraction and (B) fractional shortening in 2-month-old and 24-month-old rats; (C) ejection fraction and (D) fractional shortening in ISO-treated rats and controls. Data are means ± SEM, n = 6, **P < 0.01 vs. vehicle group. (TIF) [file pone.0182668.s002.tif]

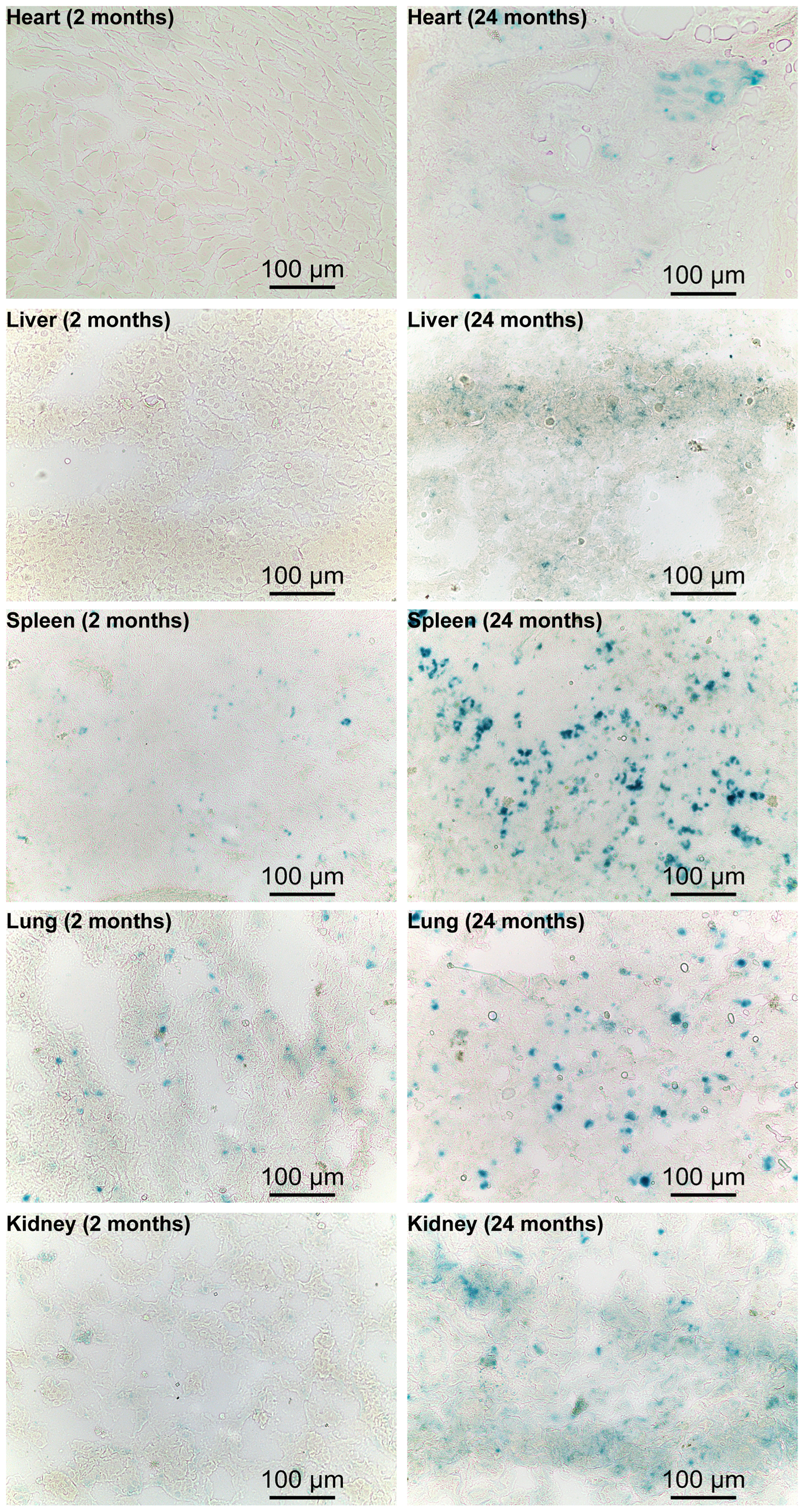

Supplement: S3 Fig — Frozen sections of heart, liver, spleen, lung and kidney from young (2-month-old) and old (24-month-old) rats were analyzed for SA-β-gal staining (n = 6). (TIF) [file pone.0182668.s003.tif]

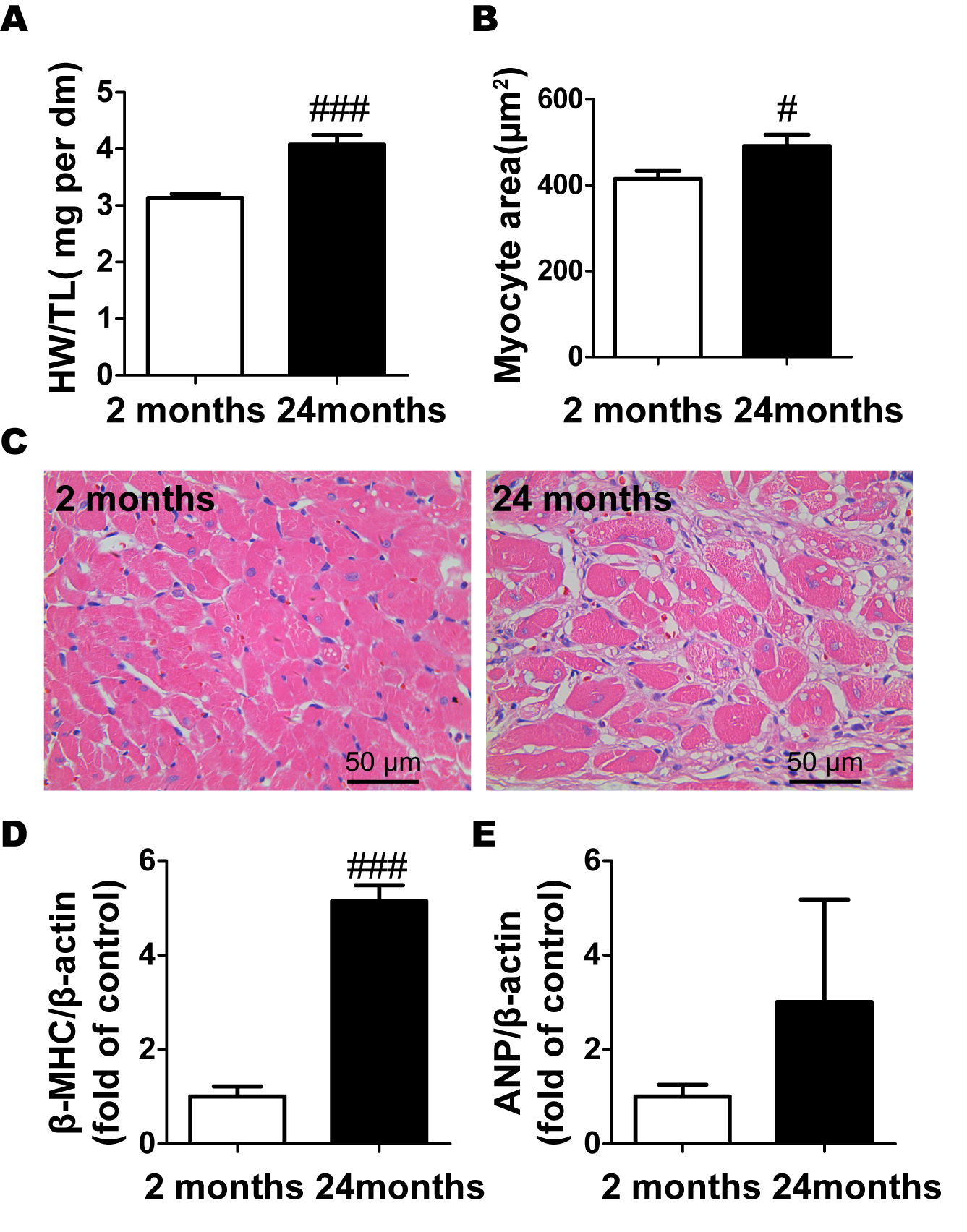

Supplement: S4 Fig — (A) HW/TL ratio in 2-month-old and 24-month-old rats. Cardiomyocyte area was evaluated by H&E staining(C) and quantified (B). The gene expression of β-MHC (D) and ANP (E) were examined with the use of quantitative RT-PCR. Data are means ± SEM, n = 6, # P < 0.05, ###P < 0.001 vs. 2 months. (TIF) [file pone.0182668.s004.tif]
